# Acquiring high expression of suilysin enable non-epidemic *Streptococccus suis* to cause streptococcal toxic shock-like syndrome (STSLS) through NLRP3 inflammasome hyperactivation

**DOI:** 10.1080/22221751.2021.1908098

**Published:** 2021-07-01

**Authors:** Lei Xu, Lan Lin, Xi Lu, Peng Xiao, Ran Liu, Meizhou Wu, Meilin Jin, Anding Zhang

**Affiliations:** a State Key Laboratory of Agricultural Microbiology, College of Veterinary Medicine, Huazhong Agricultural University, Wuhan, People’s Republic of China; b Key Laboratory of Preventive Veterinary Medicine in Hubei Province, The Cooperative Innovation Center for Sustainable Pig Production, Wuhan, People’s Republic of China; c Department of Integrated Traditional Chinese and Western Medicine, Union Hospital, Tongji Medical College, Huazhong University of Science and Technology, Wuhan, People’s Republic of China; d Key Laboratory of Development of Veterinary Diagnostic Products, Ministry of Agriculture of the People’s Republic of China, Wuhan, People’s Republic of China; e International Research Center for Animal Disease, Ministry of Science and Technology of the People’s Republic of China, Wuhan, People’s Republic of China

**Keywords:** *Streptococcus suis* (*S. suis*), streptococcal toxic shock-like syndrome (STSLS), suilysin (SLY), NLRP3 inflammasome, genome evolution

## Abstract

The epidemic *Streptococcus suis* (*S. suis*) strain [Sequence type (ST) 7] was gradually evolving from the non-epidemic ST1 strain and got the ability for high expressing of suilysin (SLY). And the high expression of SLY was required for the epidemic strain to cause NLRP3 hyperactivation, which is essential for the induction of cytokines storm, dysfunction of multiple organs, and a high incidence of mortality, the characters of streptococcal toxic shock-like syndrome (STSLS). However, it remains to be elucidated whether acquiring high SLY expression due to genome evolution was sufficient for the non-epidemic strain to cause STSLS. Here, we found that the overexpression of SLY in ST1 strain (P1/7-SLY) could obviously increase the inflammasome activation, which was dependent on NLRP3 signalling. In contrast, the strain (P1/7-mSLY) overexpressing the mutant SLY (protein without hemolytic activity) could not significantly increase the inflammasome activation. Furthermore, similar to the epidemic strain, P1/7-SLY could cause STSLS in *nlrp3*^+/+^ mice but not in *nlrp3*^−/−^ mice. In contrast, P1/7-mSLY could not cause STSLS in both *nlrp3* ^+/+^ mice and *nlrp3*^−/−^ mice. In summary, we demonstrate that genetic evolution enabling *S. suis* strain to express high level of SLY may be an essential and sufficient condition for NLRP3 inflammasome hyperactivation, which could further cause cytokines storm and STSLS.

## Introduction

*Streptococcus suis* (*S. suis*) is a major swine pathogen that is responsible for severe economic losses in the porcine industry and represents a significant threat to pig or pork-contacting peoples and immunocompetent patients [1–4]. *S. suis* infection in humans are typically sporadic, causing meningitis, sepsis, arthritis, endocarditis, and endophthalmitis, and the pooled case-fatality rate is 12.8% [5]. However, the infections unusually cause two large-scale human *S. suis* epidemics in China (the first was 25 cases with 14 deaths in Jiangsu in 1998 and the second was 204 cases with 38 deaths in Sichuan in 2005) [1,6,7]. Moreover, 97.4% fatal cases were observed with the development of streptococcal toxic-shock-like syndrome (STSLS), including the hallmarks of acute high fever, blood spots, hypotension, shock, and dysfunction of multiple organs, as well as acute death [7]. However, no superantigen responsible for toxic shock syndrome was detected in *S. suis* [7], indicating that the mechanism underlying STSLS is different from that of toxic shock syndrome. These promoted us to identify the changes of the pathogen and the host specific signalling for contribution to the STSLS.

The clinical investigations showed that STSLS patients died with cytokine storms [6]. Subsequent studies further confirmed that the induction of an inflammatory cytokine storm was essential for STSLS [8], which was further supported by the findings that the inhibition of the excessive inflammatory response with anti-inflammatory drugs improved survival against STSLS [9,10]. Our previous study indicated that the epidemic strain with high expression of SLY, the high membrane perforation activity of which caused several events, including cytosolic K^+^ efflux, and then resulted in NLRP3 inflammasome hyperactivation, which was essential for the induction of cytokines storm and STSLS in mice model [11]. The conclusion was also confirmed by another group [12]. Thus these studies supported that high expression of SLY was necessary for the epidemic strain to cause STSLS [11,12].

In fact, *S. suis* possess an open pan-genome [13,14], which was responsible for its rapid evolution of virulence and drug resistance [15], and suggested the potential of this pathogen to cause various diseases through easily acquiring exogenous genetic elements [13,16]. By comparison with non-epidemic strains, a few mobile genetic elements were found only in the Chinese epidemic strains [6,17], including a specific 89 K pathogenic island (belonging to the T4SS-mediated horizontal element), which makes the strain to show increased hemolytic activity and high virulence [18]. However, it remains to be fully elucidated whether acquiring high hemolytic activity due to genome evolution was sufficient for the non-epidemic *S. suis* strain to cause STSLS.

The recent analysis on *S. suis* genomic epidemiology indicated that the epidemic strain [Sequence type (ST) 7] was gradually evolving from the ST1 strain [16,19] and got the ability of expressing high level of SLY [18]. Thus the present study wanted to evaluate whether acquiring high expression of SLY makes ST1 strain to cause STSLS through NLRP3 inflammasome hyperactivation, and then tried to elucidate the mechanism why genome evolution enabled non-epidemic ST1 strain to cause STSLS.

## Materials and methods

### S. suis strains

The *S. suis* epidemic strain SC-19 belongs to ST7, which shows high pathogenicity in humans, mice, and pigs [10]. The isogenic mutant for *sly* (Δ*sly*) and a mutant [m*sly*(P353L)] containing a point substitution P353L were originated from strain SC-19 [11]. The *S. suis* strain P1/7, belongs to ST1, which induces only sporadic cases of meningitis and sepsis in pigs [20]. The *sly* gene and its predicted upstream promoter was cloned with primers SS2-SLY-F (5′-CGGTACCCGGGGATCCTTACTCTATCACCTCATCCGC-3′)/SS2-SLY-R (5′-CGCCAAGCTTGCATGCGTACTAAAAGCGAACTAAACAAC-3′), constructed into a *S. suis*-*E. coli* shuttle vector pSET2 [21], and then introduced into P1/7 strain to obtain the strain overexpressing functional SLY (P1/7-SLY). Similarly, the site-direct mutation of SLY (P353L) was cloned from mutant strain m*sly*(P353L) [11] with primers SS2-SLY-F/SS2-SLY-R, also constructed into pSET2, and then introduced into P1/7 strain to obtain the strain overexpressing SLY with P353L mutation (P1/7-mSLY). The plasmid pSET2 was also introduced into P1/7 strain to obtain a control strain (P1/7-Vec). Primers SLY-F (5′-GGCGAAAGGGGGATGTGCTG-3′)/SLY-R (5′-CTCATTAGGCACCCCAGGCTTTAC-3′) were designed to confirm that whether *S. suis* contained the plasmid for overexpression of SLY.

### Measurement of inflammasome activation in vitro

THP-1 cells (ATCC source) were differentiated into macrophage-like cells by treatment with 50 nM phorbol myristate acetate (PMA) (Sigma, P8139-1MG) overnight. The differentiated cells (2 × 10^6^/mL) were primed with LPS (Sigma, L4391) at 0.5 μg/mL for 4 h to induce the expression of inflammasome components, and then were infected with *S. suis* strains (2 × 10^7^/mL) or were stimulated with 5 μM ouabain (Sigma, O3125) for 2 h for the detection of inflammasome activation.

Then, 100 μL aliquots of the cell culture supernatants were collected to determine human TNF-α (invitrogen, 88-7346-88, USA) and IL-1β (invitrogen, 88-7261-88, USA) secretion levels with commercial ELISA kits. Another 100 μL aliquots of the cell culture supernatants were collected for quantifying LDH release using a CytoTox 96 NonRadioactive Cytotoxicity Assay (Promega, USA) according to the manufacturer’s instructions. The percentage of cytotoxicity was calculated based on LDH release in the total cell lysates. The cellular proteins were extracted in Laemmli sample buffer. The proteins in the supernatants were precipitated with 20% trichloroacetic acid on ice for 30 min and then washed 3 times with ice-cold acetone. After the last wash, the acetone was removed by vacuum, and the pellets were allowed to air dry for 5 min and then dissolved in Laemmli sample buffer. The proteins were subjected to immunoblot analysis with antibodies for the detection of NLRP3 (Cell Signalling, 15101S, USA), Casp1 (R&D MAB6215, USA), gasdermin D (GSDMD) (Proteintech, 66387-1-Ig, USA), or IL-1β (Proteintech, 66737-1-Ig, USA). Actin was also detected as an internal control using a specific antibody (Proteintech, 66009-1-AP, USA).

The THP-1-*nlrp3*^−/−^ cell line and its control cell line (THP-1-*nlrp3*^+/+^) [11] were also subjected to detection of inflammasome activation according to the procedure described for THP-1 cells.

### Ethics statement

The experimental infections were performed in strict accordance with the Guide for the Care and Use of Laboratory Animals Monitoring Committee of Hubei Province, China, and the protocol was approved by the Scientific Ethics Committee of Huazhong Agricultural University (Permit Number: HZAUMO-2019-044). All efforts were made to minimize the suffering of the animals.

### Experimental infections of mice

Five- to six-week-old C57BL/6 mice with similar body weights were randomly divided into groups of 8 or 10 mice in each group and challenged with 0.5 mL of *S. suis* strains (8 × 10^8^ CFU/mL) by an intraperitoneal (i.p.) injection to evaluate the pathogenicity of the different *S. suis* strains. The experimental infections were also performed on *nlrp3*^−/−^ mice (C57BL/6 background, originated from the Jackson Laboratory) to directly evaluate the effect of *nlrp3* on STSLS development.

All mice were monitored three times a day and for a total of 7 days for clinical signs. The clinical scores were assigned as follows: 0 = normal response to stimuli; 1 = ruffled coat and slow response to stimuli; 2 = respond only to repeated stimuli; 3 = non-responsive or walking in circles; and 4 = dead. Mice exhibiting extreme lethargy or neurological signs (score = 3) were considered moribund and were humanely euthanized.

In addition to the evaluation of mortality, experimental infections were also performed with mice to evaluate the overexpression of SLY on the cytokine response, blood biochemistry, and bacterial burden during *S. suis* infection. At the indicated time point of post-infection with *S. suis*, mice in each group were euthanized by carbon dioxide inhalation, and blood was collected via cardiac puncture. Fifty microliters of blood were withdrawn for bacterial load analysis. The remaining blood was used to prepare plasma for analysis of the CK (creatine kinase), ALT (alanine aminotransferase), AST (aspartate aminotransaminase), and LDH levels with a VITALAB SE Chemistry Analyser and for analysis of the IL-1β, TNF-α, IL-6, IL-17A, IL-18, IL-12p70, IL-10, and IFN-γ levels using the Electrochemiluminescence U-PLEX Biomarker Group 1 (Mouse) Multiplex Assays (MSD, 310076, USA). The brain, lung, liver, and spleen tissues were collected and fixed in 10% neutral buffered formalin and routinely processed in paraffin. Sections with a thickness of 2–3 mm were cut for haematoxylin and eosin staining for histopathologic evaluation as previously described [10].

### Bacterial load in the blood

The collected blood samples were serially diluted and then plated on Tryptic Soy Agar plates to evaluate the bacterial load.

### Statistical analysis

Unless otherwise specified, the data were analysed using two-tailed, unpaired *t*-tests. All assays were repeated at least three times, and the data are expressed as the mean ± standard deviations. For the animal infection experiments, comparisons of survival rates and clinical scores were analysed with a log-rank test or two-way RM ANOVA, respectively, using GraphPad Prism 6. For all tests, a value of *p *< 0.05 was considered significance.

## Results

### Overexpression of functional SLY enable ST1 strain (P1/7) to exhibit high hemolytic activity

*S. suis* possess an open pan-genome [13,14], which was responsible for the ST1 strain (P1/7 is a representative strain) to easily change into the epidemic strain (SC-19) due to acquiring genomic elements [16,19]. SC-19 expressed high level of SLY [18], and the high expression of SLY was necessary for the epidemic strain to cause STSLS [11]. In contrast, P1/7 expressed a relative low level of SLY [11,18]. It promoted us to evaluate whether acquiring the high expression of SLY due to genomic evolution is sufficient for the non-epidemic strain to cause STSLS.

**Figure. 1:**
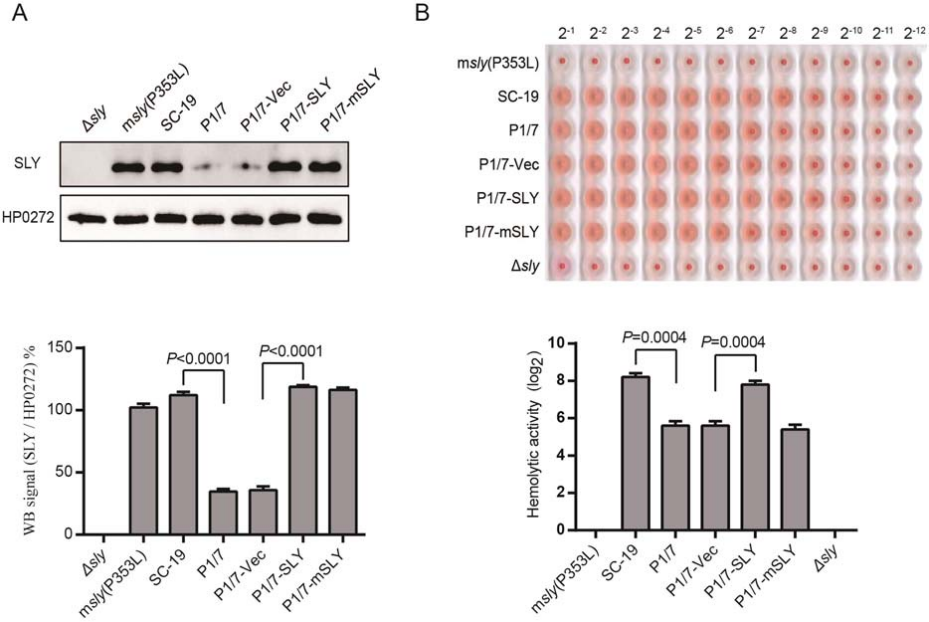
Overexpression of SLY but not mutant SLY(P353L) enables ST1 strain (P1/7) to exhibit high hemolytic activity. (A) Expression of SLY in msly(P353L), SC-19, P1/7, P1/7-Vec, P1/7-SLY, or P1/7-mSLY was detected using western blotting with a monoclonal antibody against SLY, and unrelated protein HP0272 expression was also detected as an internal control. Densitometric analysis of expression of SLY was calculated based on the Western blot signal from the SLY/HP0272. (B) Detection of hemolytic activity of SC-19-m*sly*(P353L), SC-19, P1/7, P1/7-Vec, P1/7-SLY, and P1/7-mSLY. The supernatant of *S. suis* was collected, and 1% chicken erythrocyte suspension was incubated with the supernatants for 1 h at 37°C.

Because both SC-19 and P1/7 strains contained the same *sly* gene sequence, which indicated that the differential expression of SLY was resulted from the differential regulations but not from the difference of cis-acting element. To obtain the strain with high expression level of SLY (P1/7-SLY), a *S. suis*-*E. coli* shuttle vector containing the *sly* gene element was introduced into P1/7 strain (Supplementary Figure S1A). The P1/7-SLY showed similar SLY expression and hemolytic activity to the epidemic strain SC-19, but showed significant higher level than its parent strain P1/7 (Figure 1). SLY is a member of the pore-forming cholesterol-dependent cytolysin family of toxins [22], and the P353L mutation would result in a loss of hemolytic activity while retaining the biological activity of erythrocyte aggregation [22]. As a result, the epidemic strain with mutant SLY(P353L) could not cause inflammasome hyperactivation and could not cause STSLS [11]. To provide a control for P1/7-SLY strain, the mutant SLY(P353L) gene was also constructed into *S. suis*-*E. coli* shuttle vector, and then was introduced into the P1/7 strain (P1/7-mSLY) (Supplementary Figure S1B). P1/7-mSLY showed similar SLY expression to P1/7-SLY, but showed significant lower level of hemolytic activity than P1/7-SLY (Figure 1). In addition, the empty plasmid introduced into P1/7 strain (P1/7-vec) served as another control strain, which could not alter SLY expression and hemolytic activity (Figure 1).

### Overexpression of SLY but not mSLY in P1/7 causes hyperactivation of inflammasome

**Figure 2.**
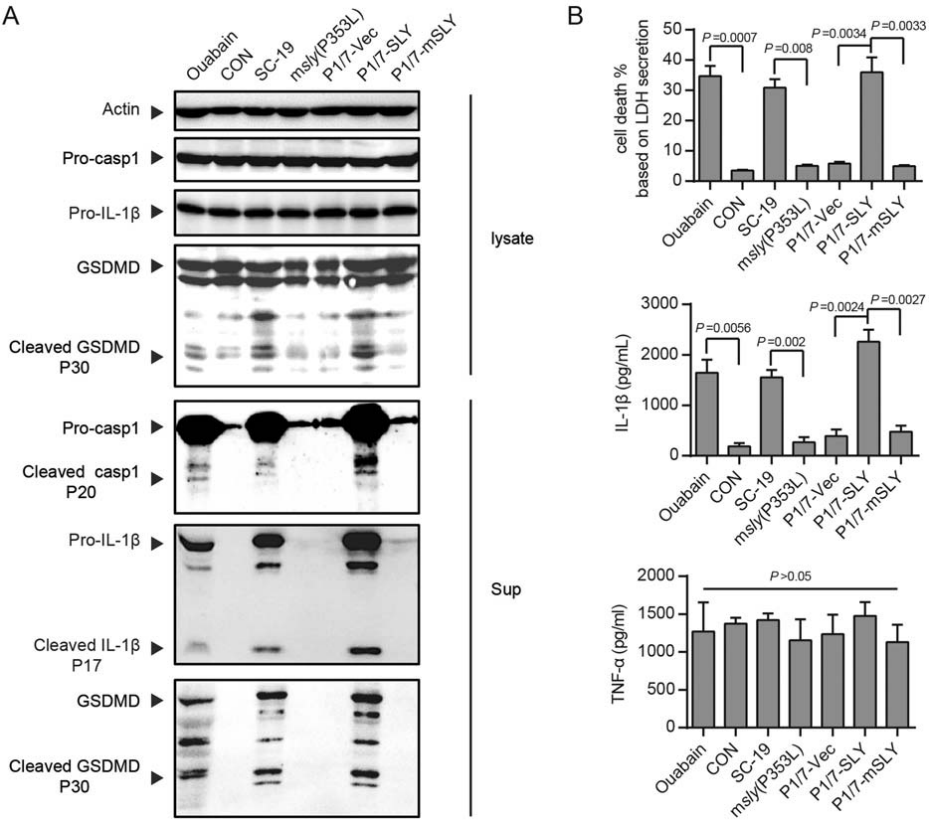
Overexpression of SLY but not mSLY in P1/7 causes hyperactivation of inflammasome. THP-1 cells were primed with LPS and then infected with *S. suis* strains SC-19, m*sly* (P353L), P1/7-Vec, P1/7-SLY, P1/7-mSLY or stimulated with ouabain. (A) The cellular proteins and the supernatants of the cell cultures were collected for the detection of Casp1, IL-1β, and GSDMD by western blot assay. (B) The concentrations of LDH, IL-1β, and TNF-α in the supernatants of THP-1 cells were detected (*n* = 3).

High expression of SLY was responsible for SC-19 to cause hyperactivation of inflammasome [11,12], indicated by the detection of high level of cleavage of pro-caspase-1, pro-IL-1β and GSDMD (Figure 2A) and high level of IL-1β secretion and cell death (Figure 2B). In contrast, SC-19 could not induce more significant TNF-α, an inflammasome-unrelated cytokine, at the detected time-point in LPS-primed THP-1 cells, in which LPS was provided to induce expression of inflammasome components and other inflammatory cytokines (e.g. TNF-α) as well (Figure 2B). These indicated that SC-19 could provide high level of the signal for the activation of inflammasome receptor and cause hyperactivation of inflammasome. However, P1/7 could not cause hyperactivation of inflammasome [11], which was also supported by the present study (Figure 2). Interestingly, the overexpression of SLY (P1/7-SLY) but not mSLY (P1/7-mSLY) in P1/7 could cause hyperactivation of inflammasome (Figure 2), indicating that acquiring high expression of functional SLY could enable ST1 strain to cause hyperactivation of inflammasome.

### Overexpression of SLY in P1/7 could cause hyperactivation of inflammasome in nlrp3^+/+^ but not in nlrp3^−/−^ monocytes

**Figure 3.**
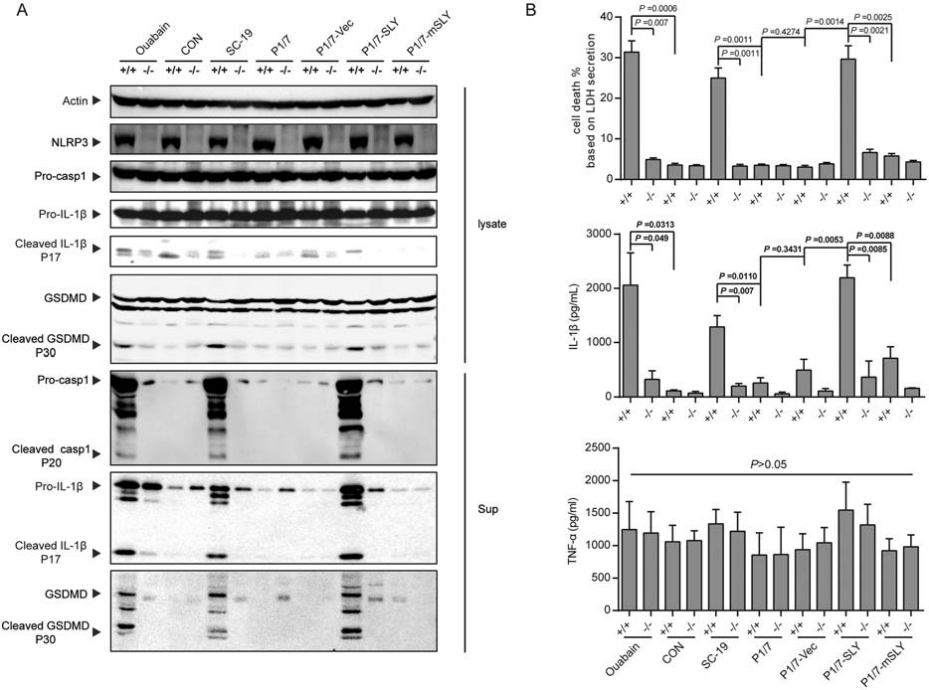
NLRP3 was critical to inflammasome activation in response to *S. suis* P1/7-SLY infection. The THP-1 *nlrp3* knockout cell line (THP-1-*nlrp3*^−/−^) and its control cell line (THP-1-*nlrp3*^+/+^) were primed with LPS and then infected with *S. suis* strains SC-19, P1/7, P1/7-Vec, P1/7-SLY, P1/7-mSLY or stimulated with ouabain. (A) The cellular proteins and the supernatants of cell cultures were collected for the detection of NLRP3, Casp1, IL-1β, and GSDMD via western blot assay. (B) The LDH, IL-1β, and TNF-α concentrations in the supernatants were also determined (*n* = 3).

The hyperactivation of inflammasome by SC-19 was dependent on the K^+^ efflux [11,12], an essential process for the recruitment of NLRP3 to the dispersed trans-Golgi network to cause K^+^-efflux-dependent NLRP3 activation [23]. High level of cleavage of pro-caspase-1, pro-IL-1β and GSDMD, high level of IL-1β secretion and cell death induced by SC-19, P1/7-SLY, and Ouabain (an NLRP3 agonist) were only observed in *nlrp*3^+/+^ but not in *nlrp*3^−/−^ monocytes (Figure 3), indicating that the hyperactivation of inflammasome by both P1/7-SLY and SC-19 was dependent on NLRP3. In contrast, no significant activation was observed by strains of P1/7, P1/7-vec, and P1/7-mSLY in both *nlrp*3^+/+^ and *nlrp*3^−/−^ monocytes (Figure 3).

Therefore, the overexpression of SLY was sufficient for ST1 strain to cause NLRP3 hyperactivation. It also suggested that acquiring high expression of SLY due to genome evolution was sufficient for the non-epidemic *S. suis* to cause hyperactivation of NLRP3 inflammasome, an essential reason for the epidemic strain to cause STSLS.

### Overexpression of functional SLY in P1/7 causes STSLS in a murine model

**Figure 4.**
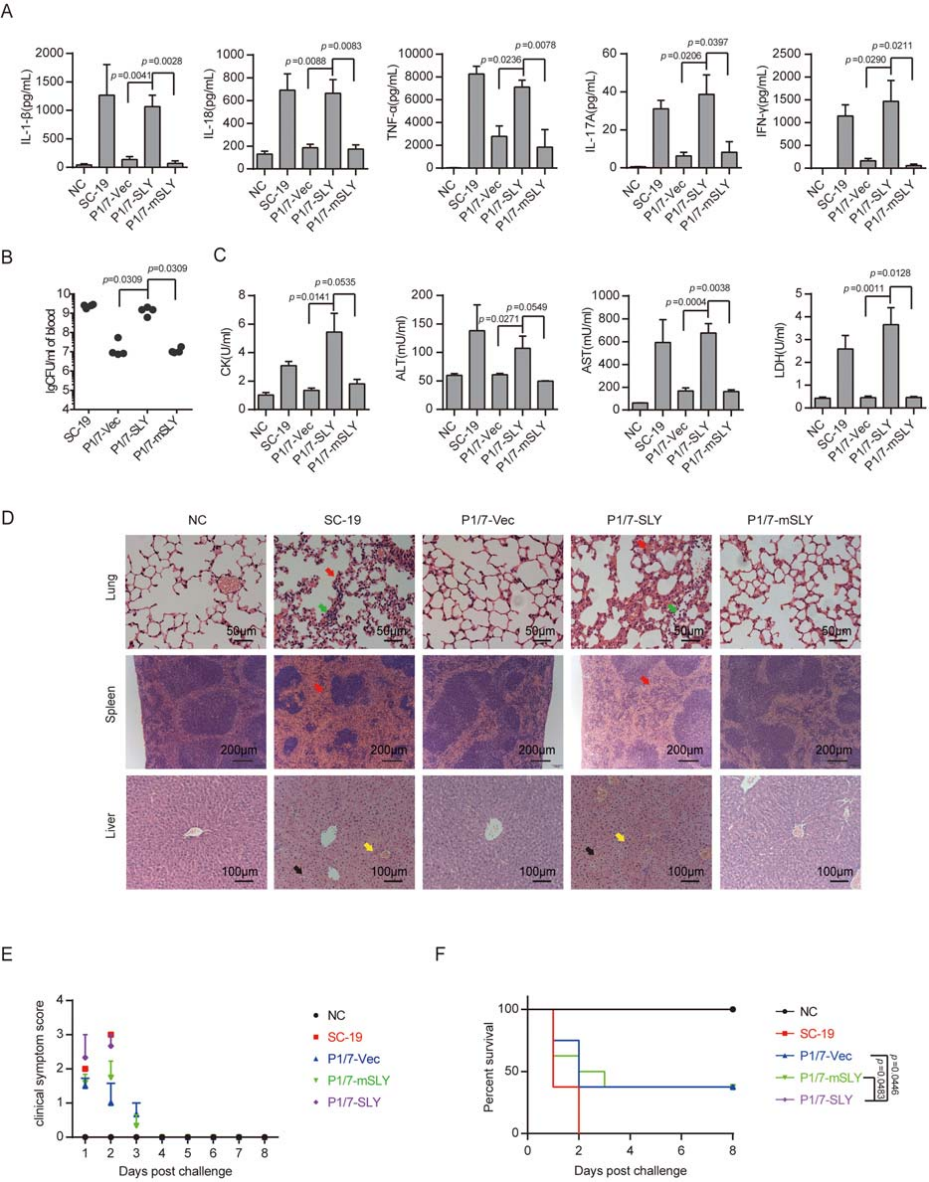
Evaluation of the role of overexpression of SLY in the non-epidemic *S. suis* strain P1/7 on STSLS with a murine model. (A) Cytokine levels in the blood at 6 h post-infection were determined (*n* = 4). (B) The bacterial load in the blood at 6 h post-infection was determined (*n* = 4). (C) Blood levels of AST, ALT, LDH, and CK at 6 h post-infection were determined (*n* = 4). (D) H&E staining of infected tissue sections from mice at 6 h post-infection with *S. suis* strains. Congestion in the lung and spleen is indicated by a “red arrow”, infiltration of inflammatory cells in the lung is indicated by a “blue arrow”, vacuolated degeneration in the liver is indicated by a “black arrow”, and necrosis in the liver is indicated by a “yellow arrow”. (E) Clinical symptom scores of mice infected with *S. suis* strains (*n* = 8). (F) Survival of mice infected with *S. suis* strains (*n* = 8). Error bars represented the mean ± standard deviations.

STSLS is characterized by high bacterial burden, an inflammatory cytokine storm, multi-organ dysfunction, and ultimately acute host death [6,7]. The previous study indicated that the epidemic strain SC-19 but not P1/7 strain could cause STSLS in a murine model [11,12], which were also observed in the present study (Figure 4).

Similar to the epidemic strain, P1/7-SLY could induce an acute and extremely high inflammatory cytokine response (Figure 4A), high bacterial burden (Figure 4B), and high CK, ALT, AST, and LDH levels in the blood (Figure 4C), resulting in evident injury in multiple organs, such as severe congestion and dense infiltration of inflammatory cells in the lung, severe congestion in the spleen, and severe vacuolated degeneration and necrosis in the liver (Figure 4D). In addition, all mice infected with P1/7-SLY presented with severe clinical signs and died within 2 days (*n* = 8) (Figure 4E,F). In contrast, P1/7-vec strain could not cause an inflammatory cytokine storm, multi-organ dysfunction, and high mortality (Figure 4).

In addition, P1/7-mSLY could also overexpress similar high level of SLY to P1/7-SLY, but showed significant lower level of hemolytic activity (Figure 1). It was interesting that P1/7-mSLY could not induce high levels of the inflammasome-regulated pro-inflammatory cytokines IL-1β and IL-18 or the downstream effectors, including IL-17A and IFN-γ (Figure 4A), and it did not result in high levels of ALT, AST, LDH and CK in the blood (Figure 4C). Furthermore, 40% of the mice infected with P1/7-mSLY survived, and much lower clinical signs and alleviated organ damage were observed during the trail (Figure 4D–F). These data further confirmed that the membrane perforation activity but not the side effect due to overexpression of SLY in P1/7 was required for high level of inflammasome activation and the development of STSLS.

Therefore, the overexpression of functional SLY could enable P1/7 stain to cause an inflammatory cytokine storm, multi-organ dysfunction, and ultimately acute host death, the characters of STSLS. It also suggested that acquiring high expression of functional SLY was sufficient for the epidemic strain to cause STSLS.

### Overexpression of SLY in P1/7 could cause STSLS in nlrp3^+/+^ but not in nlrp3^−/−^ mice

Because the development of STSLS by SC-19 was dependent on the activation of NLRP3 [11,12], and the high level of inflammasome activation by SC-19 and P1/7-SLY was also mainly through NLRP3 *in vitro* (Figure 2), it is necessary to evaluate the contribution of NLRP3 activation by P1/7-SLY to STSLS.

**Figure 5.**
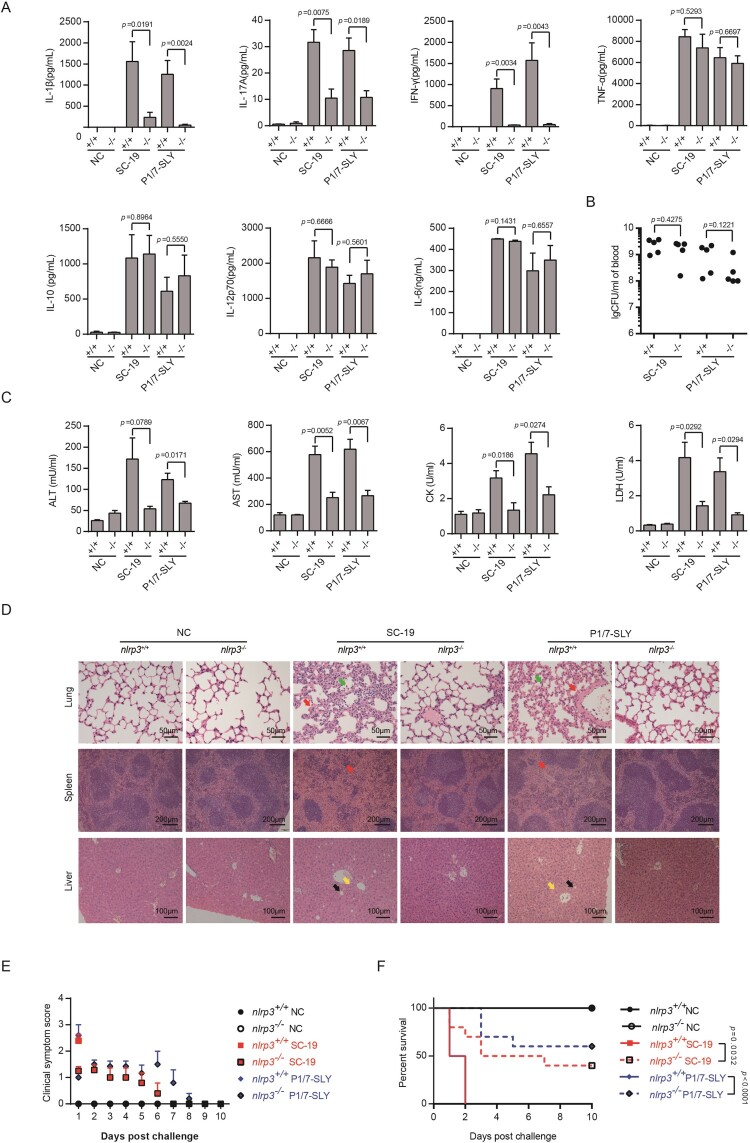
*nlrp3* was required for STSLS caused by *S. suis* P1/7 overexpressing SLY. (A) Cytokine levels in the blood at 6 h post-infection were determined (*n* = 5). (B) The bacterial load in the blood at 6 h post-infection was determined (*n* = 5). (C) Blood levels of AST, ALT, LDH, and CK at 6 h post-infection was determined (*n* = 5). (D) H&E staining of infected tissue sections from mice at 6 h post-infection with *S. suis* strains. Congestion in the lung and spleen is indicated by a “red arrow”, infiltration of inflammatory cells in the lung is indicated by a “blue arrow”, vacuolated degeneration in the liver is indicated by a “black arrow”, and necrosis in the liver is indicated by a “yellow arrow”. (E) Clinical symptom scores of mice infected with *S. suis* strains (*n* = 10). (F) Survival of mice infected with *S. suis* strains (*n* = 10). Error bars represented the mean ± standard deviations.

In coincidence with the previous report [11,12], the characters of STSLS disappeared from the infection by SC-19 on *nlrp3*^−/−^ mice (Figure 5). Similar effects were observed for infection of P1/7-SLY on *nlrp3*^−/−^ mice, it induced significantly decreased level of IL-1β, IL-17A, and IFN-γ comparing to the infection on *nlrp3*^+/+^ mice (Figure 5A), while the bacterial burden in the blood did not significantly decrease at the given time point (Figure 5B). The infection on *nlrp3*^−/−^ also significantly decreased levels of CK, ALT, AST, and LDH in the blood (Figure 5C), alleviated injury in multiple organs (Figure 5D), reduced clinical signs (Figure 5E), and promoted host survival (Figure 5F). It was not surprising that the control strain P1/7-mSLY and P1/7 strain containing the control empty vector (P1/7-vec) could not cause STSLS in *nlrp3*^+/+^ and *nlrp3*^−/−^ mice (Supplementary Figure S2). These results suggested that the mechanism for P1/7-SLY causing STSLS was similar to that by SC-19, which were mainly induced through hyperactivation of NLRP3 inflammasome.

## Discussion

*S. suis* has an open genome, which enables the pathogen to easily evolve into various phenotypes [13,14], and the Chinese highly virulent strain is such an example [6,17]: it caused two unusual outbreaks of human *S. suis* diseases with high mortality due to STSLS [7]. Although the comparative genomic studies suggested that acquiring genetic elements was responsible for the highly virulence [13,16], the mechanism for STSLS required more experimental data to be elucidated. Our previous study indicated that high expression of SLY was necessary for the epidemic strain to activate NLRP3 inflammasome, which was essential for STSLS [11]. However, the direct data were still lacked to confirm whether acquiring the ability of expressing high level of SLY in non-epidemic strain due to genetic evolution was sufficient to cause STSLS. Because the epidemic ST7 strain was evolved from ST1 strain [16,19], the present study directly evaluated whether the overexpression of SLY in the ST1 strain P1/7 could cause STSLS. From the present study, we found that acquiring high SLY expression was sufficient for P1/7 strain to cause STSLS through NLRP3 inflammasome hyperactivation. In combination with these studies, we can give a full explanation that acquiring high expression of SLY in the ST1 strain was sufficient for NLRP3 inflammasome hyperactivation, which could further cause cytokines storm and STSLS.

The essential role of NLRP3 inflammasome hyperactivation on the development of STSLS could not controvert the opinion that inflammasome activation played critical functions in pathogen defence [24]. It was further supported by a recent study on the inflammasome activation by SLY-negative *S. suis* indicating that inflammasome activation was important for controlling bacterial infection [25]. However, no significant difference was observed on the survival rate and clinical symptoms of infection by P1/7 strain and P1/7-SLY in *nlrp3*^−/−^ mice, while P1/7-SLY but not P1/7 strain caused development of STSLS in *nlrp3*^+/+^ mice (Figure 5 and Supplementary Figure S2), these also supported our previous conclusion that hyperactivation of NLRP3 was essential for STSLS development [11]. Therefore, inflammasome activation, as a one type of innate immune system, could discriminate microbes from “self” by canonical type inflammasome (which could directly or indirectly recognize the pathogenic effects by NLRP3, NAIP/NLRC4, NLRP1, AIM2, or Pyrin receptors) or non-canonical inflammasome (which directly recognized cytosolic LPS by caspase-4, caspase-5, and caspase-11) and then destroy invading microbes [24]. However, hyperactivation of inflammasome could be undoubtedly harmful to the host [11,26]. This might be the reason that the researchers tried to evaluate the role of inflammasome activation on severe COVID-19, which could also cause the patients to present cytokines storm and multiple-organs failure [27,28], and tried to identify the inflammasome as a potential target for treatment [29,30]. The present study supported the potential targeting inflammasome for treatment of severe diseases characterized by cytokines storm and multiple-organs failure through the inflammasome hyperactivation.

The present study indicated that NLRP3 inflammasome could be activated mainly by SLY, but it does not mean that only NLRP3 inflammasome could be activated by *S. suis*. Except for NLRP3 inflammasome, *S. suis* could also activate AIM2, NLRP1, and NLRC4 types of inflammasome through lipoproteins [25] or potential peptidoglycan [31]. Our previous and the present study also supported the opinion that *S. suis* could still induce a low level of mature IL-1β in *nlrp3*^−/−^ THP-1 cells (Figure 3) [11]. Moreover, the level of activation of these types of inflammasome was not high and may probably contribute to controlling the infection [25,32]. Therefore, hyperactivation of NLRP3 inflammasome was the essential reason for STSLS, which was mainly the result from high expression of SLY.

In addition, SLY played various roles on the pathogenesis of *S. suis* in addition to causing NLRP3 inflammasome activation through cytosolic K^+^ efflux signalling [11,12]. For example, it was confirmed to be involved in resistance to complement-mediated killing [33,34] and complement C3aR/C5aR-mediated monocytes chemotaxis [35]; it was also the main stimulus for TNF-α production independently of its membrane perforation ability [36] and was involved in the invasive infection and virulence of *S. suis* [37–40]. These functions could be confirmed by the test that the overexpression of functional SLY enable P1/7 to be more resistant to the host clearance (Figure 4). Furthermore, the present study also indicated that the hemolytic activity of SLY was responsible for STSLS, because the overexpression of mutant SLY into ST1 strain could not cause STSLS (Figure 4). This might be the reason why so many potential drugs targeting SLY could attenuate pathogenicity of the epidemic *S. suis* [41–46].

**Figure 6.**
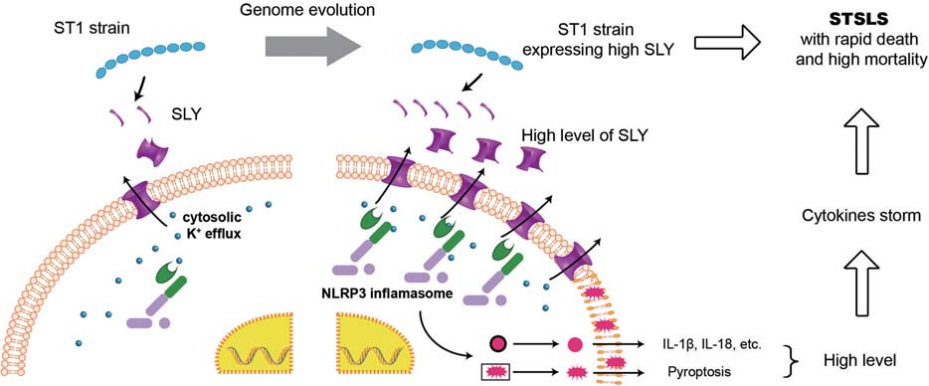
Acquiring high expression of SLY enabled non-epidemic *S. suis* to cause STSLS through NLRP3 inflammasome hyperactivation. It also provided an explanation why the epidemic *S. suis* strain, which evolved from ST1 strain and expressed high level of SLY, could suddenly cause a cytokines storm and STSLS.

In conclusion, the present study demonstrated that the overexpression of functional SLY but not mutant SLY in ST1 strain is sufficient for causing STSLS. It indicated that high expression of SLY in the ST1 strain due to genetic evolution was sufficient for NLRP3 inflammasome hyperactivation, which could further cause a cytokines storm and STSLS. Therefore, the present study also provided an explanation why the epidemic *S. suis* strain expressing high level of SLY could suddenly cause STSLS (Figure 6).
